# Supplementary material for: A High-Quality Genome Assembly of Striped Catfish (Pangasianodon hypophthalmus) Based on Highly Accurate Long-Read HiFi Sequencing Data
Source: Genes (Basel). 2022 May 22;13(5):923. doi: 10.3390/genes13050923 (PMC9141817; doi:10.3390/genes13050923)
Supplement: Supplementary file 1 [file genes-13-00923-s001.zip › genes-1712001-supplementary.pdf]

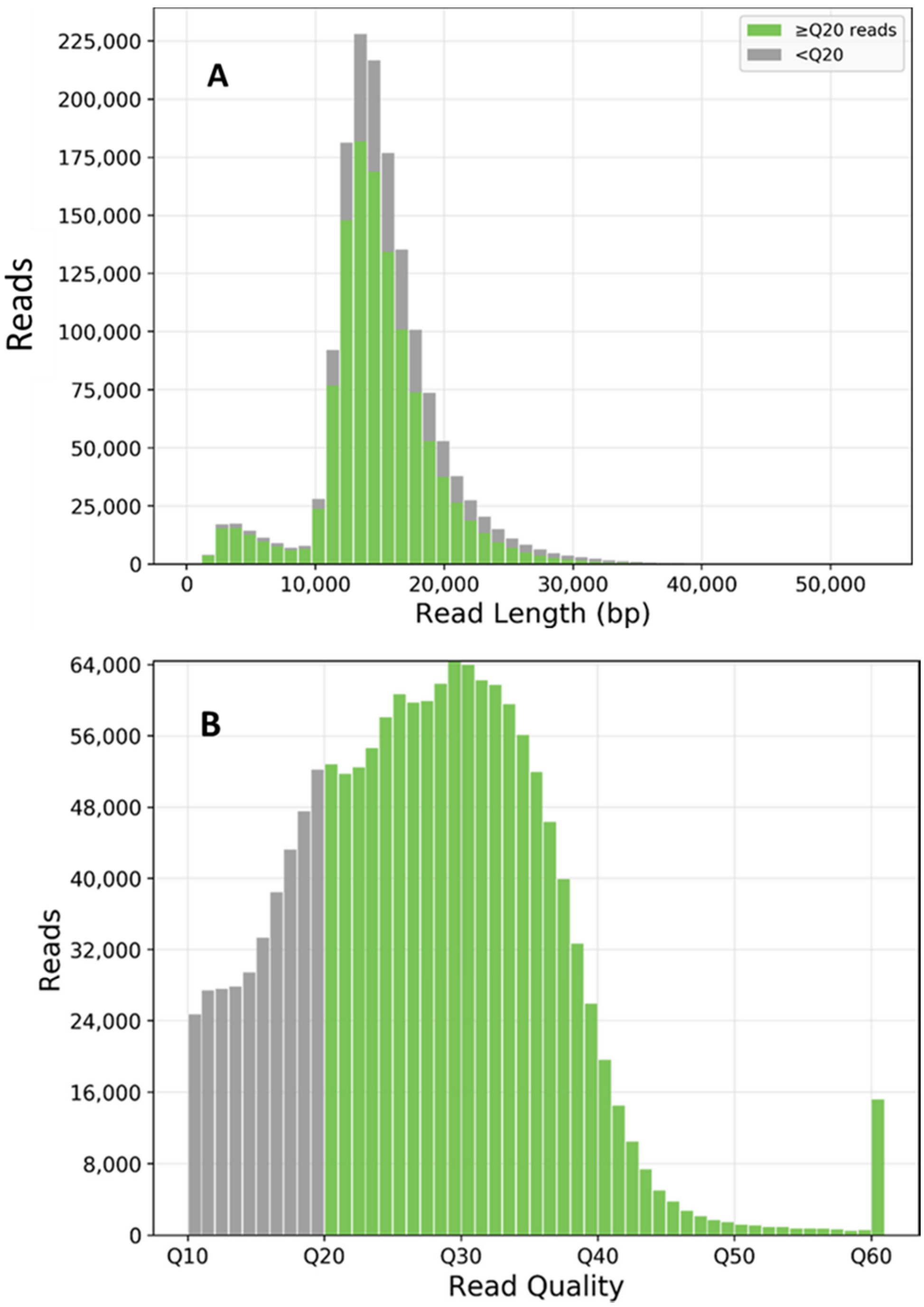

Figure S1: Distribution of read length (A) and read quality (B). Used HiFi sequences (Q ≥ 20) are shown in green.

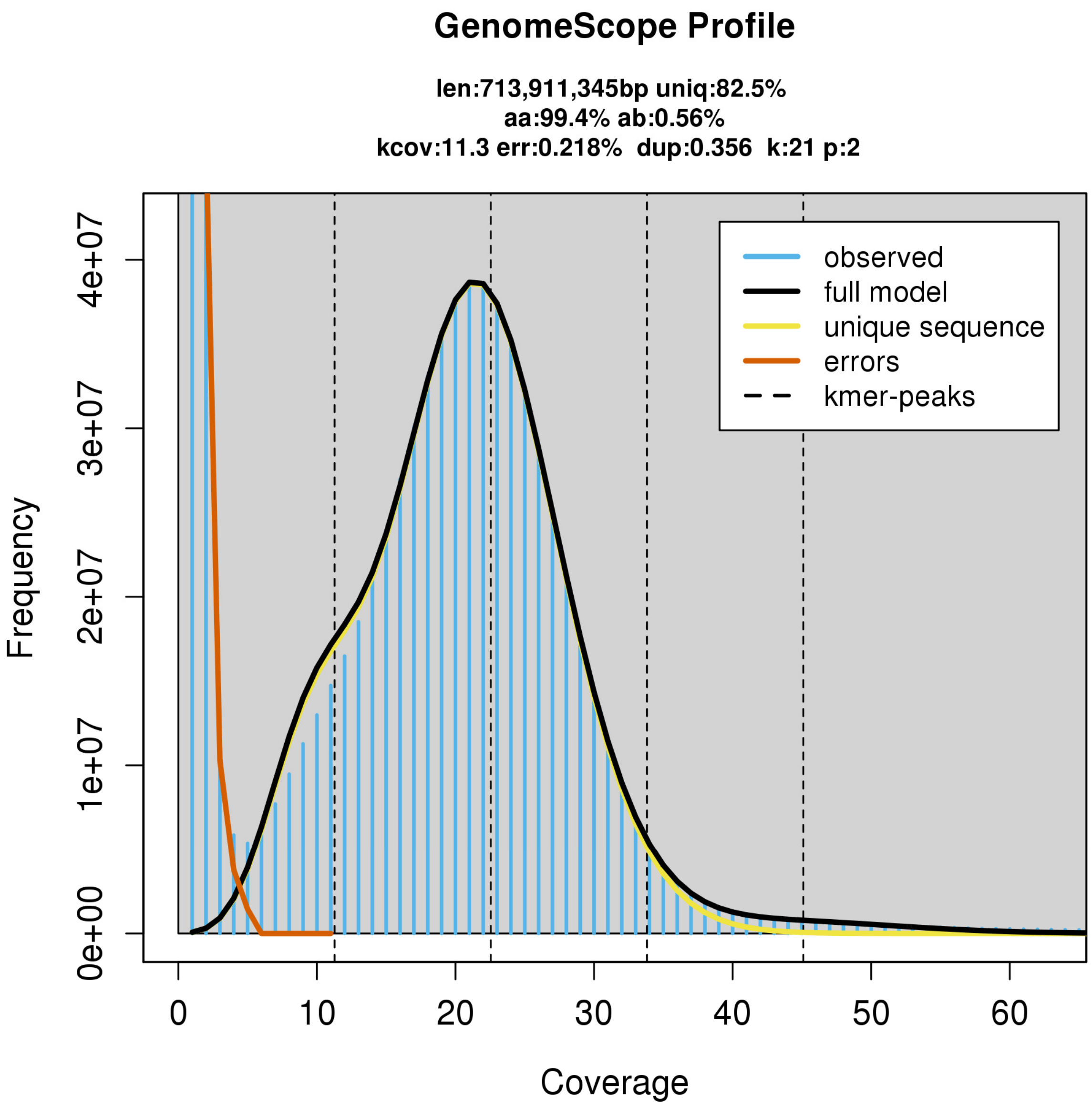

Figure S2: A *k-mer* analysis of the genome sequencing reads for the striped catfish using GenomeScope

Table S1 Repetitive elements in the striped catfish genome

TE Class	Family	Sub-family	De novo			Homology		
			Count	Length (bp)	Percentage	Count	Length (bp)	Percentage
Class I (Retroelements)	Non-LTR elements	SINEs:	20,832	31,13,943	0.39	50584	6755978	0.86
		Penelope	1,757	615,424	0.08	6402	1467082	0.19
		LINEs:	98,550	30,509,760	3.87	121,145	29,469,338	3.74
		CRE/SLACS	0	0	0.00	0	0	0.00
		L2/CR1/Rex	76,961	24,726,066	3.14	77,327	20,030,762	2.54
		R1/LOA/Jockey	1,567	82,3571	0.10	1,918	412,240	0.05
		R2/R4/NeSL	1,219	376,738	0.05	649	270,429	0.03
		RTE/Bov-B	8,119	1,813,291	0.23	19,762	3,853,265	0.49
		L1/CIN4	3,687	935,167	0.12	3,221	1,146,312	0.15
	LTR elements	BEL/Pao	2,006	781,888	0.10	1,030	399,154	0.05
		Ty1/Copia	0	0	0.0	1,642	513,400	0.07
		Gypsy/DIRS1	42,978	14,309,769	1.82	45,254	15,858,477	2.01
		Retroviral	2,639	924,326	0.12	6,469	1,185,074	0.15
		Total LTR elements:	87,491	23,554,747	2.99	79,867	21,805,013	2.77
Total Retroelements (a):			206,873	57,178,450	7.25	251,596	58,030,329	7.36
Class II (DNA transposons)		hobo-Activator	99,579	17,622,531	2.24	122,119	19,242,281	2.44
		Tc1-IS630-Pogo	193,506	53,485,756	6.78	230,862	59,455,614	7.54
		En-Spm	0	0	0.00	0	0	0.00
		MuDR-IS905	0	0	0.00	0	0	0.00
		PiggyBac	2,069	67,2161	0.09	4,871	838,840	0.11
		Tourist/Harbinger	20,401	4,866,723	0.62	29,947	5,121,870	0.65
		Other (Mirage, P-element, Transib)	1,608	321,938	0.04	1,660	460,487	0.06
Total DNA transposons (b):			442,919	101,790,693	12.91	576,633	115,072,113	14.60
Rolling-circles (c)			11,569	2,998,325	0.38	18437	3986467	0.51
Unclassified (d):			694,670	112,360,030	14.25	680699	125,718,307	15.95
Total interspersed repeats (a+b+c+d)				271,329,173	34.42		298,820,749	37.90
Small RNA:			3,756	2,166,067	0.27	5,071	1,558,812	0.20
Satellites:			1,817	497,961	0.06	11,591	2,308,540	0.29
Simple repeats:			573,469	28,230,949	3.58	520,916	22,917,129	2.91
Low complexity:			45,375	3,133,402	0.40	40,587	2,517,469	0.32
Total bases masked:				308,355,877	39.11		331,921,288	42.10

Table S2 Overview of simple sequence repeats (SSRs)

Cutoffs and Range				
SSR Type	Set of repeating bases	Repetition number for the set	Example	Total Length
Mono nucleotide Repeats (p1)	1	>= 10 bases	AAAAAAAAAAAA	>=10 t0 Any length
Di nucleotide Repeats (p2)	2	>= 6 Pairs	CACACACACACACA	>=12 to Any length
Tri nucleotide Repeats (p3)	3	>= 5 Sets	AACAACAACAACAAC	>=15 to Any length
Tetra nucleotide Repeats (p4)	4	>= 5 Sets	AAACAAACAAACAAACAAAC	>=20 to Any length
Penta nucleotide Repeats (p5)	5	>= 5 Sets	AAATGAAATGAAATGAAATGAAATG	>=25 to Any length
Hexa nucleotide Repeats (p6)	6	>= 5 Sets	AAATATAAATATAAATATAAATATAAATAT	>=30 to Any length

Cutoffs and Range<br>Statistics	Count
Total number of sequences examined	381
Total size of examined sequences (bp)	788,355,903
Total number of identified SSRs	960,574
Number of SSR containing sequences	377
Number of sequences containing more than 1 SSR	376
Number of SSRs present in compound formation	320,010
p1	315,319
p2	510,826
p3	73,996
p4	53,670
p5	4,726
p6	2,037
